# Structural Diversity and Initial Oligomerization of PrP106–126 Studied by Replica-Exchange and Conventional Molecular Dynamics Simulations

**DOI:** 10.1371/journal.pone.0087266

**Published:** 2014-02-19

**Authors:** Lulu Ning, Jingjing Guo, Qifeng Bai, Nengzhi Jin, Huanxiang Liu, Xiaojun Yao

**Affiliations:** 1 State Key Laboratory of Applied Organic Chemistry and Department of Chemistry, Lanzhou University, Lanzhou, China; 2 Gansu Computing Center, Lanzhou, China; 3 School of Pharmacy, Lanzhou University, Lanzhou, China; 4 State Key Lab for Quality Research in Chinese Medicines, Macau Institute for Applied Research in Medicine and Health, Macau University of Science and Technology, Taipa, Macau, China; German Research School for Simulation Science, Germany

## Abstract

Prion diseases are marked by cerebral accumulation of the abnormal isoform of the prion protein. A fragment of prion protein composed of residues 106–126 (PrP106–126) exhibits similar properties to full length prion and plays a key role in the conformational conversion from cellular prion to its pathogenic pattern. Soluble oligomers of PrP106–126 have been proposed to be responsible for neurotoxicity. However, the monomeric conformational space and initial oligomerization of PrP106–126 are still obscure, which are very important for understanding the conformational conversion of PrP106–126. In this study, replica exchange molecular dynamics simulations were performed to investigate monomeric and dimeric states of PrP106–126 in implicit solvent. The structural diversity of PrP106–126 was observed and this peptide did not acquire stable structure. The dimeric PrP106–126 also displayed structural diversity and hydrophobic interaction drove the dimerization. To further study initial oligomerization of PrP106–126, 1 µs conventional molecular dynamics simulations of trimer and tetramer formation were carried out in implicit solvent. We have observed the spontaneous formation of several basic oligomers and stable oligomers with high β-sheet contents were sampled in the simulations of trimer and tetramer formation. The β-hairpin formed in hydrophobic tail of PrP106–126 with residues 118–120 in turn may stabilize these oligomers and seed the formation oligomers. This study can provide insight into the detailed information about the structure of PrP106–126 and the dynamics of aggregation of monomeric PrP106–126 into oligomers in atomic level.

## Introduction

Prion diseases are fatal neurodegenerative disorders that include, for instance, bovine spongiform encephalopathy, scrapie of goat and sheep, Creuzfeldt–Jacob disease and Kuru of humans [1]–[3]. This group of diseases is characterized by the aggregation of misfolded forms of prion protein [4]. In contrast with the cellular prion protein (PrP^C^), the abnormal prion protein (PrP^Sc^) is partially protease K-resistant and displays marked property to accumulate into insoluble aggregates and amyloid fibrils [1], [3], [5]. PrP^C^ and PrP^Sc^ share the identical primary structure [6] but compared to PrP^C^, PrP^Sc^ is characterized by the dominantly increased β-sheet content [4], [7]. Structural studies show that the normal prion protein is composed of two structurally distinct domains: an extended N-terminal region (residues 23–125) and a well-defined C-terminal region (residues 126–231) consisting of three α-helices and two short in-register β-sheets [8]–[12]. The structure of the infectious forms PrP^Sc^ is less characterized due to its noncrystalline and oligomeric nature. The mechanism underlying the transition from the cellular prion protein to its scrapie form has not been fully elucidated.

Previous researches have shown that a synthetic peptide corresponding to residues 106–126 of prion protein exhibits many characteristics typical of PrP^Sc^ [13]–[15]. For instance, this peptide causes apoptosis of nerve cells and induces the proliferation and hypertrophy of astrocytes and stimulates the microglia [13]–[15]. It is of interest that the toxicity of PrP106–126 depended on cellular synthesis of PrP^C^ [16], which is in accordance with the observation that neuronal cell death in scrapie infection *in vivo* requires the expression of PrP^C^ [17]. It is also reported that the selective deletion of residues 23–88 does not inhibit the conversion from PrP^C^ to PrP^Sc^, while the removal of residues 108–121 or 122–140 along with residues 23–88 prevents PrP^Sc^ formation, which indicate that the region including residues 106–126 may play a key role in the structural conversion of PrP^C^ and PrP^Sc^ [18]. Furthermore, PrP106–126 shows a tendency to aggregate into amyloid fibrils that is partially resistant to digestion with protease [19]. Thus, PrP106–126 can be used as a relevant model to investigate the PrP^Sc^-mediated cell apoptosis and fibrils formation.

The primary structure of PrP106–126 is comprised of an N-terminal hydrophilic region (KTNMKH-) and a long hydrophobic tail (-MAGAAAAGAVVGGLG). It has been shown that PrP106–126 displays remarkable structural polymorphism, acquiring different secondary structure in different conditions. The chemical physical conditions such as pH, solvent composition and ionic strength affect the secondary structure of PrP106–126 [20]–[22]. Though many efforts have been made to study the structure of PrP106–126 [20], [21], [23], [24], detailed conformations of PrP106–126 are not fully elucidated due to the impressed polymorphism of the peptide in solution. Due to its aggregating nature and its high tendency to form complex fibril, the structure of PrP106–126 is difficult to obtain by X-ray crystallography. Furthermore, the structural information about PrP106–126 have been obtained mostly by studies using NMR and CD, both of which only investigate the properties of bulk solution, which may contain monomer and various oligomers. Molecular dynamics simulations can provide an appropriate method to study the structure of isolated PrP106–126. Becker et al carried out multiple molecular dynamics simulations and observed that the helix of PrP106–126 and its A117V mutant transit to coil spontaneously and the β-sheet conformation of wild type PrP106–126 is sensitive to pH and less stable in acid environment [22]. Bowers et al performed replica exchange molecular dynamics simulations to examine the structure of monomeric PrP106–126 and the result suggested that the β-hairpin is the dominant structure of PrP106–126 in solution [25], which is different from the structural polymorphism of PrP106–126 suggested by other researches [20], [22], [26]. Though molecular dynamics simulations of PrP106–126 have been performed earlier, the structure of monomeric PrP106–126 is still controversial and the nature of the folding of PrP106–126 has not been thoroughly elucidated.

Furthermore, the nonfibrillar oligomers of PrP106–126 have been proposed to be cytotoxic, which have been found to either impair the cellular membranes [27] or form ion channels [28]–[30]. However, the structural information about the nonfibrillar amyloid oligomers of PrP106–126 and the molecular mechanism of PrP106–126 oligomers formation are largely unknown, although several structural studies have gained insights into this issue [25], [31], [32]. Early events on the pathway to fibril formation are hard to study due to the transient nature of oligomers. Molecular dynamics simulations also have provided a convenient method to get clues about the interpeptide association. Molecular dynamics simulations have been carried out to probe the mechanism of early oligomerization of other peptides successfully such as Aβ peptides [33]–[42], β_2_-Microglobulin [43], [44], h-IAPP [45] and other short peptide [46]–[49] which all can form amyloid.

In this study, we performed replica exchange molecular dynamics simulations to analyze the conformational spaces of monomeric PrP106–126. Then, based on the most representative coil extracted from the structures of monomer, replica exchange molecular dynamics simulations on the formation of the dimer of PrP106–126 peptides were carried out. Furthermore, to probe the aggregation process of trimer and tetramer, conventional molecular dynamics simulations were performed.

## Materials and Methods

### Simulation setup

The starting conformation of the monomer was fully extended and constructed in Discovery Studio 2.5.5 [50]. Though the majority of experimental studies on PrP106–126 have been performed on the unblocked peptide, we removed the charges on the termini of PrP106–126 by acetylating and amidating its N-terminal Lys and C-terminal Gly, respectively. We have made such a choice based on the notion that the AcPrP106–126NH_2_ peptide is more similar to the sequence inserted in prion protein than the uncapped.

**Figure 1:**
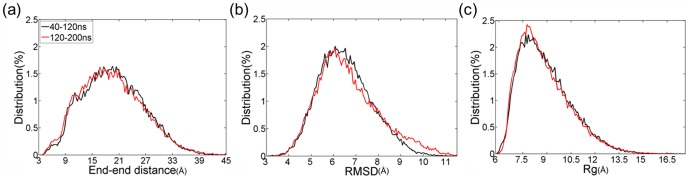
Validation of convergence of the REMD simulations using two time intervals: 40–120 and 120–200 ns at 301.98 K. (a) End-to-end distance distributions of PrP106–126. (b) Distributions of backbone RMSD. (c) Distributions of backbone radius of gyrations.

In our simulations of dimer, trimer and tetramer formation, the most populated coil from replica exchange molecular dynamics simulations was taken as the initial structure. The peptides were placed randomly and the distances between each other were no less than 25 Å. To mimic the cellular crowded environment, the whole system was confined in an imaginary sphere so that when the atoms were beyond the given distance from the center of the sphere the harmonic force centered at this position would prohibit the atoms from coming out of the sphere. The radius from the center of the sphere was maintained as 45 Å for the dimer system, 50 Å for the trimer and 55 Å for the tetramer. Provided that the end to end distance of PrP106–126 is no more than 45 Å (shown as Fig. 1a), the radius allowed the fully extension of PrP106–126 by providing sufficient space.

### Simulation protocols

In the study of the monomeric PrP106–126, replica exchange molecular dynamics simulations were carried out. The strategy of REMD [51] is to simulate multiple replicas concurrently but at different temperature. In periodic interval, the attempts to swap the temperatures of neighbouring replicas are made based on Metropolis Monte Carlo criterion [52]. Such exchanges are to facilitate the simulations to escape from local minimum energy states, which is a distinctive advantage of REMD method. To define the temperature distributions of replicas, a web server for generating temperatures for REMD-calculation was employed [53]. 16 replicas of monomer were set up with their temperatures exponentially distributed (270.00, 285.59, 301.98, 319.10, 337.25, 356.36, 376.46, 397.63, 419.91, 443.34, 467.98, 493.95, 521.24, 549.14, 580.15 and 611.92 K). The AMBER 10 [54] suite was used in the REMD simulations and ff99SB force field [55]–[57] was employed in combination with the modified Generalised Born solvent model developed by Onufriev et.al [58]. In the replica exchange molecular dynamics simulations of the monomer, 2500 steps of steepest decent minimization and 2500 steps of conjugate gradient minimization were performed to eliminate unnatural collision. For the first 1 ns, molecular dynamics simulations were carried out without replica exchange to equilibrate each system at its target temperature. Then, exchanges between neighboring replicas were attempted every 1000 steps. The time step was 2 fs with SHAKE [59] algorithm employed to constrain the bond involved in hydrogen atoms. The overall exchange rate among replicas was ∼40%. The simulation time was 200 ns for each replica resulting in an accumulative simulation time of 3.2 µs.

As we know, the nonfibrillar oligomers of PrP106–126 have been proposed to be cytotoxic. However, the structural information about the nonfibrillar amyloid oligomers of PrP106–126 and the molecular mechanism of PrP106–126 oligomers formation are largely unknown. To study the initial oligomerization of PrP106–126, REMD simulations on dimer have been carried out. 24 replicas were set up with their temperatures exponentially distributed (270.00, 280.01, 290.37, 301.6, 312.2, 323.55, 335.37, 347.57, 360.20, 373.25, 386.75, 400.70, 415.11, 430.01, 445.41, 461.33, 477.77, 494.78, 512.35, 530.51, 549.28, 568.68, 588.72 and 609.43 K). Simulations protocols of the dimer were identical to that of the monomer with an accumulative simulation time of 4.8 µs.

To study the process of oligomerization of PrP106–126, 1 µs MD simulations were performed for trimer and tetramer formation respectively in 270 K. The experimental researches have indicated that low temperature is better to capture the oligomers and low temperatures are often adopted to prepare oligomers of amyloid-prone peptides [60]–[62]. Furthermore, computational studies also used low temperature to investigate the property of oligomers [63]. Thus, a low temperature of 270 K was used to more efficiently sample oligomers. The protocols of conventional molecular dynamics simulations were similar to these of the individual replica of REMD.

### Analysis methods

The trajectory of replica exchange molecular dynamics simulations at 301.98 K for monomer and 301.06 K for dimer which are close to room temperature was selected to be analyzed. All the analyses of replica exchange molecular dynamics simulations took no account of the first 40 ns. The convergence of REMD was rigorously checked by calculating of the distributions of end-to-end distance, backbone RMSD, backbone radius of gyration (Rg) in two time intervals 40–120 and 120–200 ns. The end-to-end distance was the distance between the Cα atoms of the 106th residue and the 126th residue. Backbone RMSD was calculated with the initial structure as reference structure. We used the K-means algorithm in the MMTSB toolset [64] to cluster the conformations sampled in the REMD simulations of monomeric PrP106–126. The secondary structures were all determined by STRIDE [65] algorithm built in VMD [66]. Side chain contacts were defined by the 6.5 Å cutoff for the distance between their mass centers. If there was at least one side chain contact between two peptides, the two peptides were considered to be aggregated.

## Results and Discussion

### Replica exchange molecular dynamics simulations of monomeric PrP106–126

#### Ensemble-averaged secondary structure of each residue in monomeric PrP106–126

Prior to characterizing the atomic structures of monomer, convergence of REMD simulations was checked. As shown in Fig. 1, there is little difference in the distributions of end-to-end distance, backbone RMSD and backbone Rg in the two intervals 40–120 ns and 120–200 ns. It can be seen that REMD simulations of monomer have converged to equilibrium state in 200 ns.

**Figure 2:**
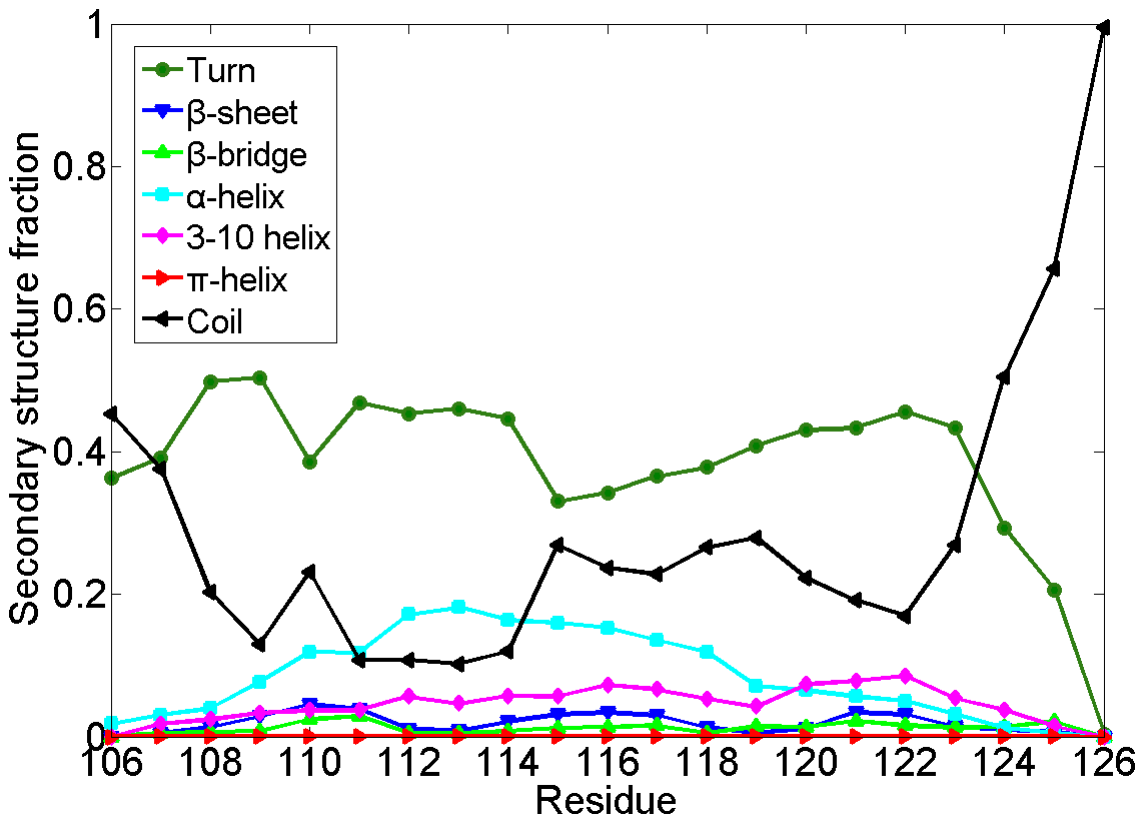
Ensemble-averaged secondary structure fraction for each residue of PrP106–126. Seven secondary structure elements are turn, β-bridge, α-helix, 3–10 helix, π-helix, coil and β-sheet, respectively.

To investigate the secondary structure features of local segments, the ensemble-averaged population of each residue in PrP106–126 acquiring one kind of STRIDE secondary structure was investigated. As shown in Fig. 2, the residues in PrP106–126 peptide adopt unstructured turn and coil predominately, which is consistent with the observation that the blocked PrP106–126 exhibit random coil structure in neutral aqueous solution [20], [22], [26]. It is noteworthy that the residues 112–119 have a high α-helix propensity while residue 119–123 displays a notable 3–10 helix tendency. β-sheet is also observed though in a low contents. By contrast, for all the residues in PrP106–126 there is low tendency to form β-bridge and π-helix. In general, PrP106–126 is tended to adopt random coil structure and its central region is more inclined to acquire well-defined structures compared to the two termini.

#### Characterizing the conformational ensemble for PrP106–126

**Figure 3:**
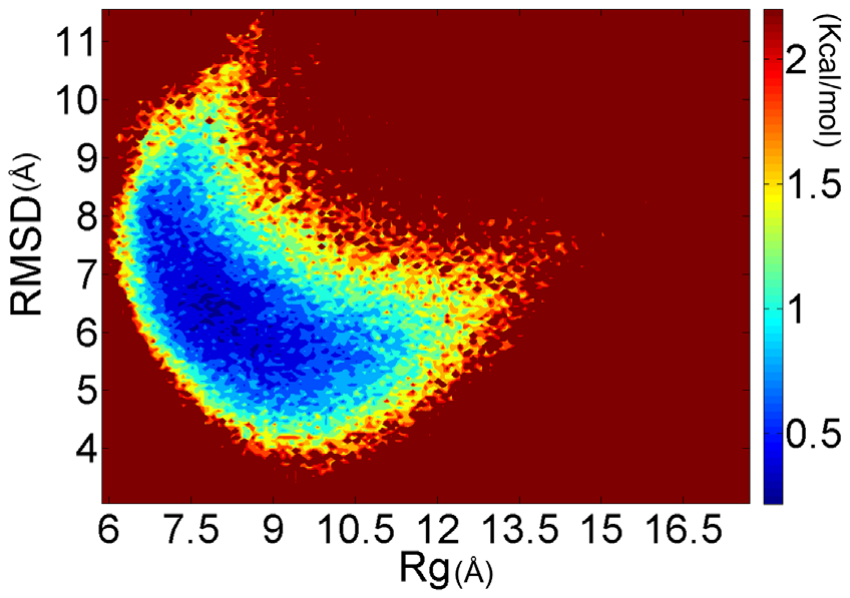
The potential of mean force of PrP106–126 at 301.98 K with backbone RMSD and backbone radius of gyration (Rg) as reaction coordinates.

**Figure 4:**
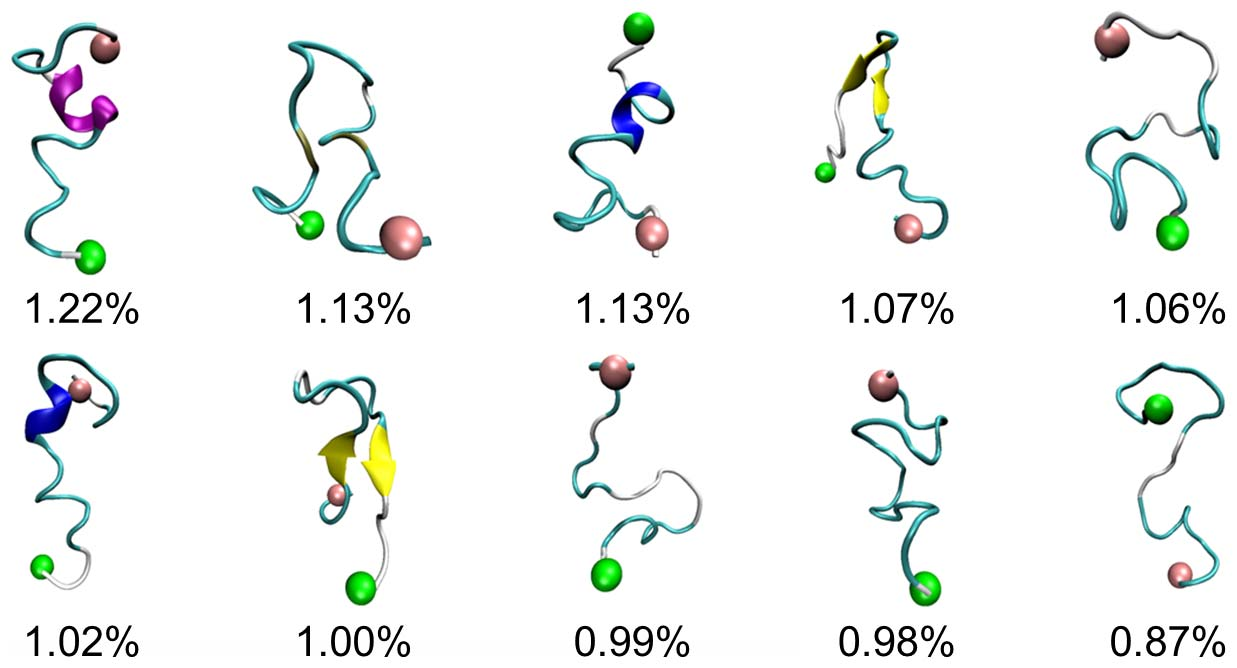
The top ten most populated representative conformations of PrP106–126. The N-terminal region and the C-terminal region are labeled by pink and green balls respectively. α-helices are in purple, extended β-sheets in yellow, 3–10 helices in blue, turns in cyan and coils in white.

To probe the global, collective conformational space of PrP106–126, potential of mean force was calculated and projected along backbone RMSD and backbone radius of gyration. As shown in Fig. 3, the colormap of PMF shows a rather featureless contour and no well-separated basins, which indicates that the monomeric conformational ensemble of PrP106–126 displays significant diversity and no native state is indentified. This is consistent with the notion that PrP106–126 peptides are disordered intrinsically. It has been reported that the peptide homologous to the 106–126 residues of human prion protein in the acetylate and amide form at their N- and C-terminal displays a predominantly random structure in aqueous solution [26]. In Fig. 4, the structures of the ten most populated conformations in PrP106–126 ensemble were pictured. Backbone RMSD was used as similarity measure with cutoff 5 Å. The proportion of each cluster is small, with their populations of only 1.22% 1.13%, 1.13%, 1.07%, 1.06%, 1.02%, 1.00%, 0.99%, 0.98%, and 0.87% respectively (Fig. 4.). Moreover, their structures are various, suggesting that PrP106–126 displays significant structural diversity. However, in the study of Bowers et al [25], monomeric PrP106–126 presented high population of β-hairpin structure. The difference may primarily come from the different treatment of termini (unblocked termini was applied in ref.25). Another reason might arise from different force field. Different force fields impact the results of simulations differently [67]. For example, CHARMM 22 overestimates the stability of helical structure [68]. AMBER force field ff96 shows bias favoring extended β-structure [55], [69], [70]. The influence of force fields on early formed amyloid oligomers has also been reported [71]. AMBER ff99 strongly favors helical structures for the monomer and does not predict any β-sheet structure for the dimer and trimer. GROMOS favors turn-coil conformations in the monomer and a very high population of extended β-sheet structures in the dimer and trimer. OPLS force field displays an intermediate tendency between AMBER and GROMOS. In our work, ff99SB was used. Compared to ff99 which biases toward helical structure [71], AMBER ff99SB provides much improved proportions of helical versus extended structures and corrected the glycine sampling and should also perform well for β-turn structures [55].

**Figure 5:**
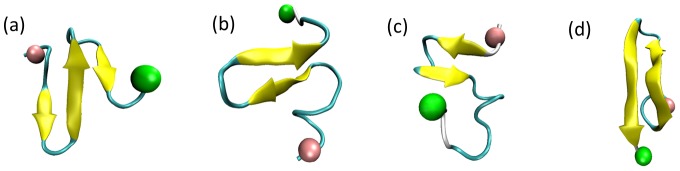
Representative β-hairpins and β-sheets from REMD of monomeric PrP106–126.

Molecular dynamics simulations of PrP fragment similar to PrP106–126 and full-length PrP provided some structural insights of PrP106–126 [72]–[78]. The study by Derreumaux suggested that PrP106–126 had a clear preference for β-sheet structure [72]. Daidone et al proposed that PrP109–122 assumed a β-hairpin structure [73]. Through the simulation of the full-length PrP90–231, the tail of prion explored a β-hairpin from residue 120–130, a β-sheet consisting 114–113 and 109–107 and random states. As for PrP109–213, residues 111–114, 120–123 of PrP109–213 participated in three-stranded β-sheet which was found in equilibrium with β-hairpin and random states [75]. Additionally, some mutations [75], [76] and low pH [77], [78] can incline the fragments including PrP106–126 to acquired β-stranded structure. In summary, the residues involved in PrP106–126 displayed high propensity to acquire β-sheets or β-hairpins. β-sheets or β-hairpins have been also sampled in REMD of monomeric PrP106–126 though with low probability about 6% in total (Fig. 5). The different probability to display β-structure of PrP106–126 in different studies may arise from different sampling methods, different force fields and different contexts adopted by these researches.

Since PrP106–126 possesses similar pathological and physicochemical characteristics to PrP^Sc^ and can be used as PrP^Sc^-mimicking peptide, our simulation results about PrP106–126 can provide some insights for the full-length PrP90–231 and can be partly transplanted to PrP90–231. In PrP90–231, residues 106–126 are highly conserved and the conformational change of this region plays important role in the conversion from PrP^C^ to PrP^Sc^. Although this shorter fragment may not be fully representative of the full-length prion, the studies of PrP106–126 may help explore the fundamental aspects of misfolding and oligomerization of prion protein.

#### The residue contact map of monomeric PrP106–126

**Figure 6:**
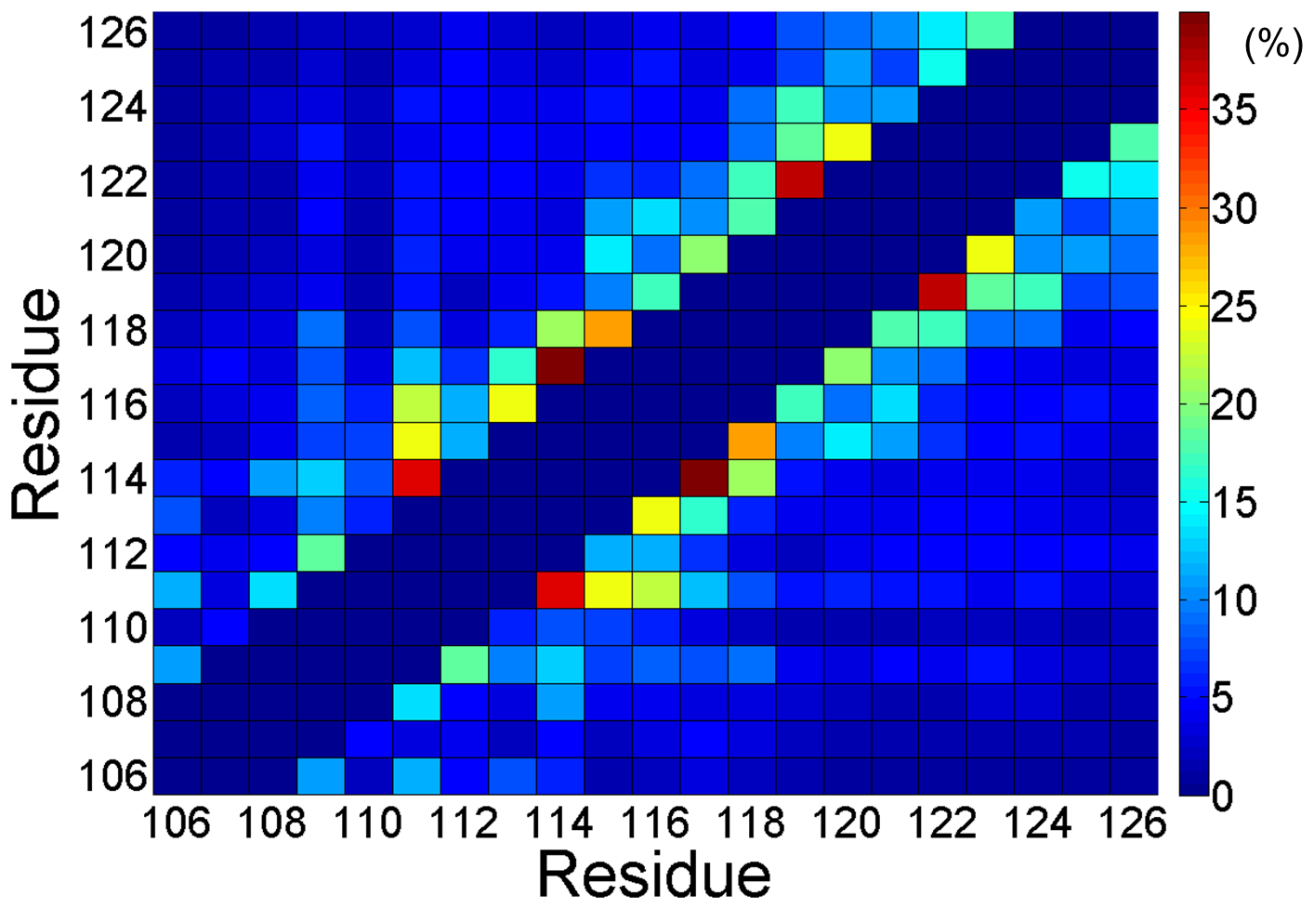
Ensemble-averaged contact map for PrP106–126. The (i,i), (i,i±1), and (i,i±2) are not included and result in the dark blue diagonal as shown in this figure.

To probe the key interaction of folding process of PrP106–126 monomer, the contact map was analyzed over the last 160 ns (Fig. 6). Frequent (i,i+3) and (i,i+4) contacts indicated that residues 111–124 are tended to form helix, in accordance with the secondary structure contents as shown in Fig. 2. It is noteworthy that except the strong contacts forming in the helix-forming region there is no distinct regions with considerable inter-residue contacts in the contact map. This observation can be elucidated by the fact that PrP106–126 is flexible and does not display a well-define structure in the most of the simulation time. There is no stable hydrophobic core or insisting salt bridge forming in capped monomeric PrP106–126. In a word, for monomeric PrP106–126, there is no dominant driving force such as hydrophobic interaction or electrostatic interaction to keep this peptide in a stable structure, which may partly elucidate the intrinsic disorders of PrP106–126.

### PrP106–126 oligomerization: dimer, trimer and tetramer formation from monomers

#### Replica exchange molecular dynamics simulations of dimeric PrP106–126

**Figure 7:**
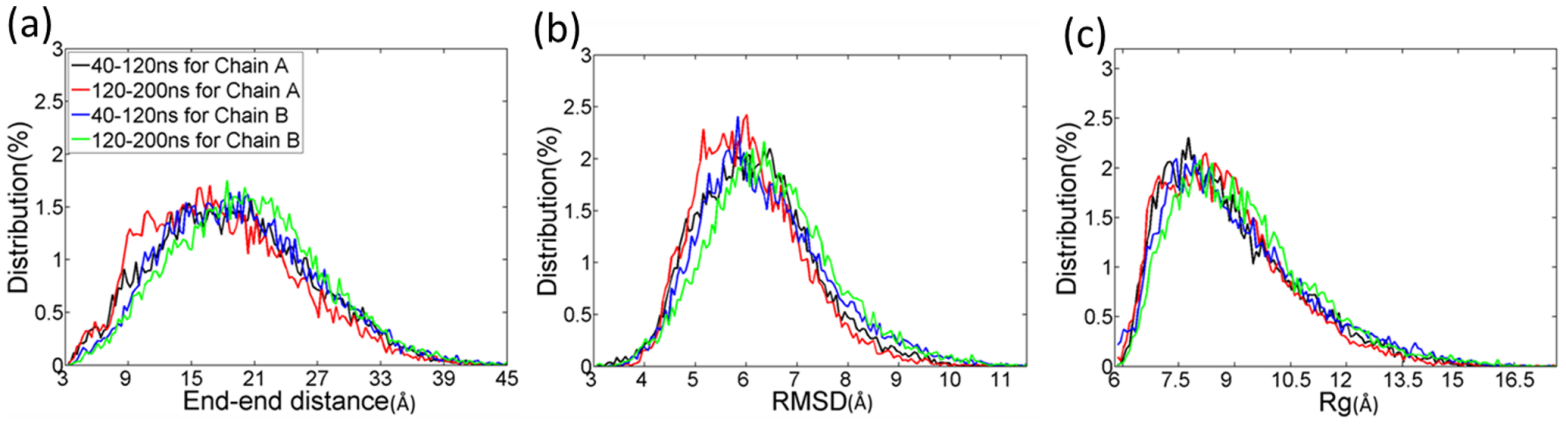
Convergence of the REMD simulations of dimeric PrP106–126.

To check the convergence of replica exchange molecular dynamics simulations of dimeric PrP106–126, same criteria as monomer were applied. The respective distributions of end-to-end distance, RMSD and Rg of the two chains in different time intervals are roughly the same as shown in Fig. 7, which indicates that REMD sampled almost the same conformational spaces in the two intervals.

**Figure 8:**
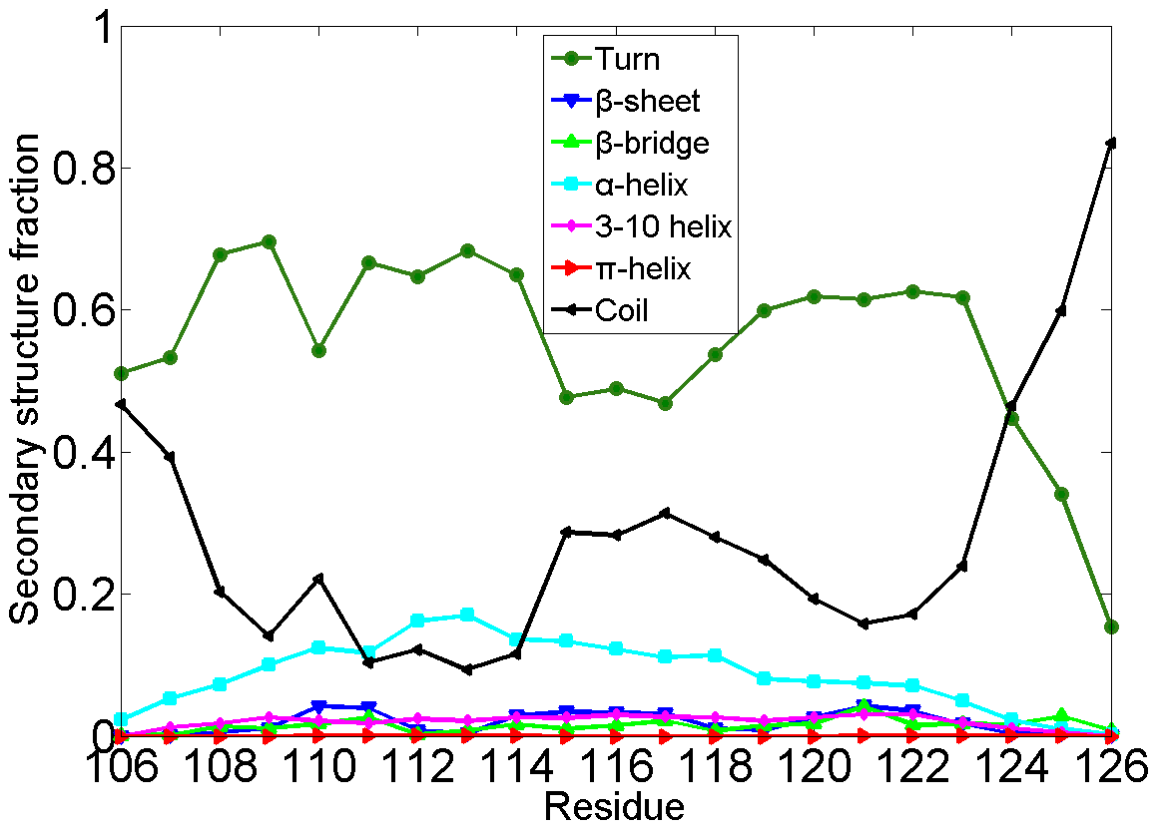
Averaged secondary structure contents for the residues included in dimeric PrP106–126. The secondary structure contents were averaged over the two homologous chains.

To investigate the structural property of dimer, we extracted the dimers in 40–200 ns and calculated the averaged secondary structures for residues. As shown in Fig. 8, the residues in dimeric PrP106–126 assumed unstructured coil and turn predominantly, which are similar to those of monomeric PrP106–126. Compared the monomer, the dimeric Residues in hydrophobic tail of PrP106–126 experience slightly increase of β-bridge and β-sheet contents. The 3–10 helix contents decreased evidently, special for the hydrophobic tail. Our REMD simulations suggest that ensemble-averaged secondary structures of PrP106–126 monomers and dimers are similar.

**Figure 9:**
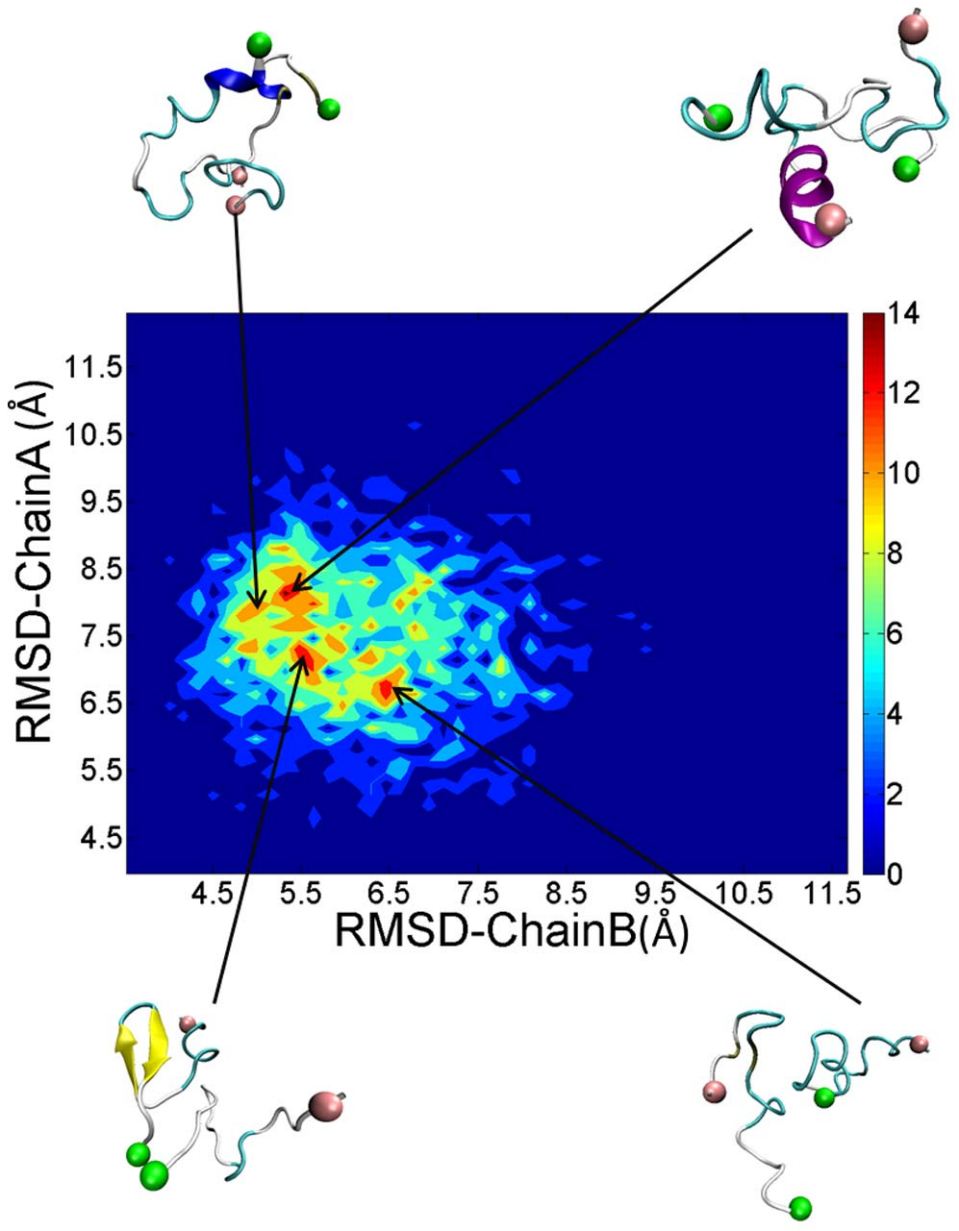
Distributions and representative structures of dimeric PrP106–126.

To access how the structures of each monomer changes when included inside a dimer, we calculated the structural distributions of each chain according to their RMSDs of each conformation. As shown in Fig. 9, there is a rich diversity of structures with several local basins observed. Representative structures corresponding to different local basins displayed different secondary structure. The orientations of the two peptides are also diverse. However, the dimers also have something in common. The positively charged N-termini were placed away from each other and the hydrophobic tails were in close contact with each other. The advantage of this arrangement was that it reduced the electrostatic repulsion and enhances hydrophobic interaction, which resulted in favorable structures.

**Figure 10:**
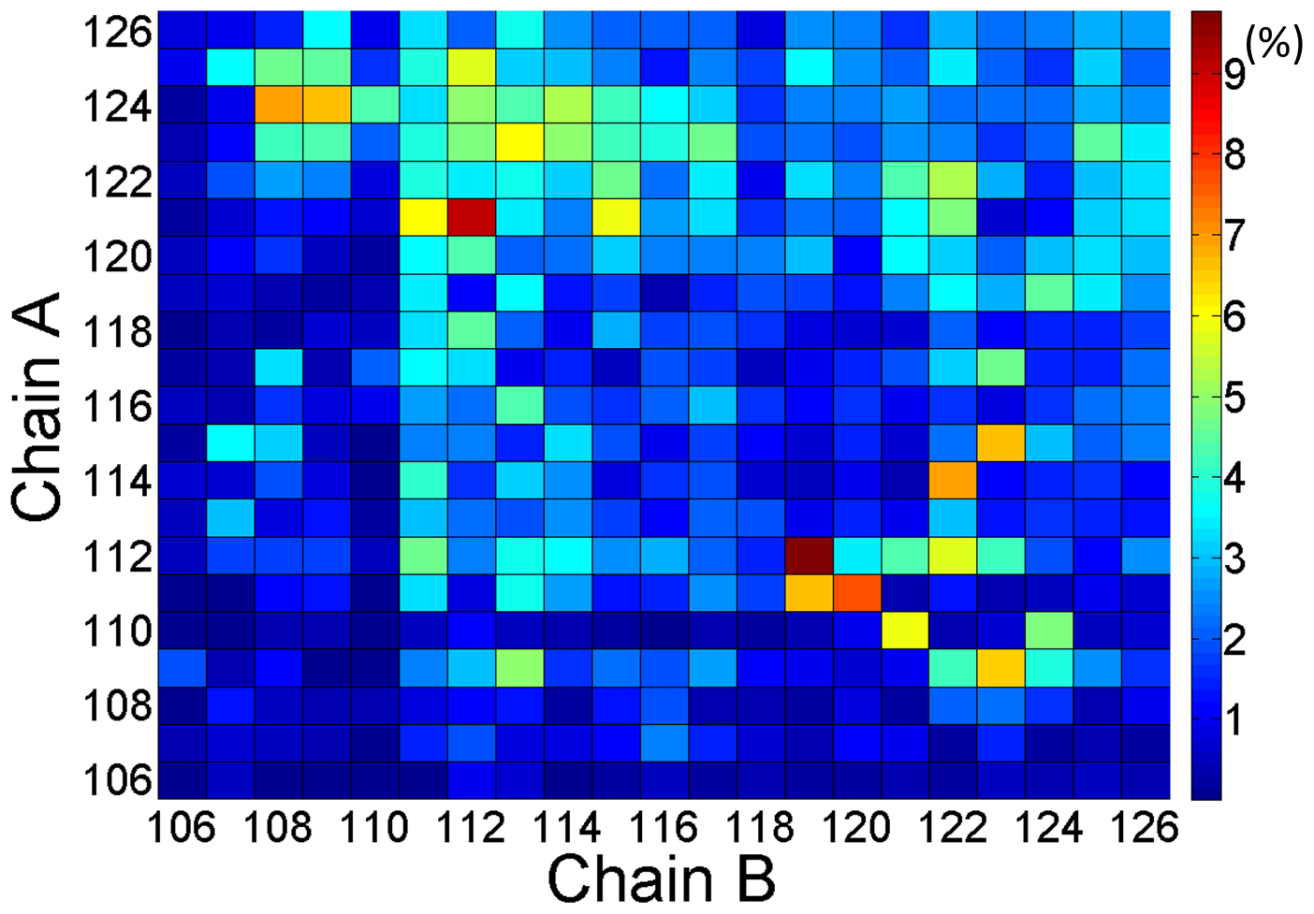
Interchain contact map of PrP106–126.

To visualize the interchain contacts that occur in PrP106–126 dimerization, we plot in the Fig. 10 contact maps for pairs of amino acids side chains. The contact maps are the ratios of accumulative contacts numbers of each residue in 40–200 ns to the total number of the dimers sampled in REMD simulations. As shown in Fig. 10, the side chain contacts mainly occur in hydrophobic residues and positively charged residues displayed much weaker propensity to contact with other residues. The residues in the central part of PrP106–126 show low probability to interact with the central residues in the other peptide. The region of the highest probability of contacts can be found between the regions H111-A115 and G119–G124. Interestingly, residues H111 and M112 display much stronger propensity to contact with other residues. Previous researches have suggested that the two residues play key roles in the aggregation of PrP106–126 [79], [80]. Our results show that the contacts presenting in PrP106–126 dimer are not specific, which indicates that the dimers are not characterized by any particular conformational state.

#### The process of oligomers formation investigated by conventional molecular dynamics simulations

REMD simulations on the dimer provided insights into the conformational characteristics and the interaction modes of the dimers, furthermore, the simulations of process of oligomers formation have also been carried out to provide direct information of initial aggregation of PrP106–126. To study the initial step of oligomerization of PrP106–126, the aggregation process of trimer and tetramer formation were simulated.

**Figure 11:**
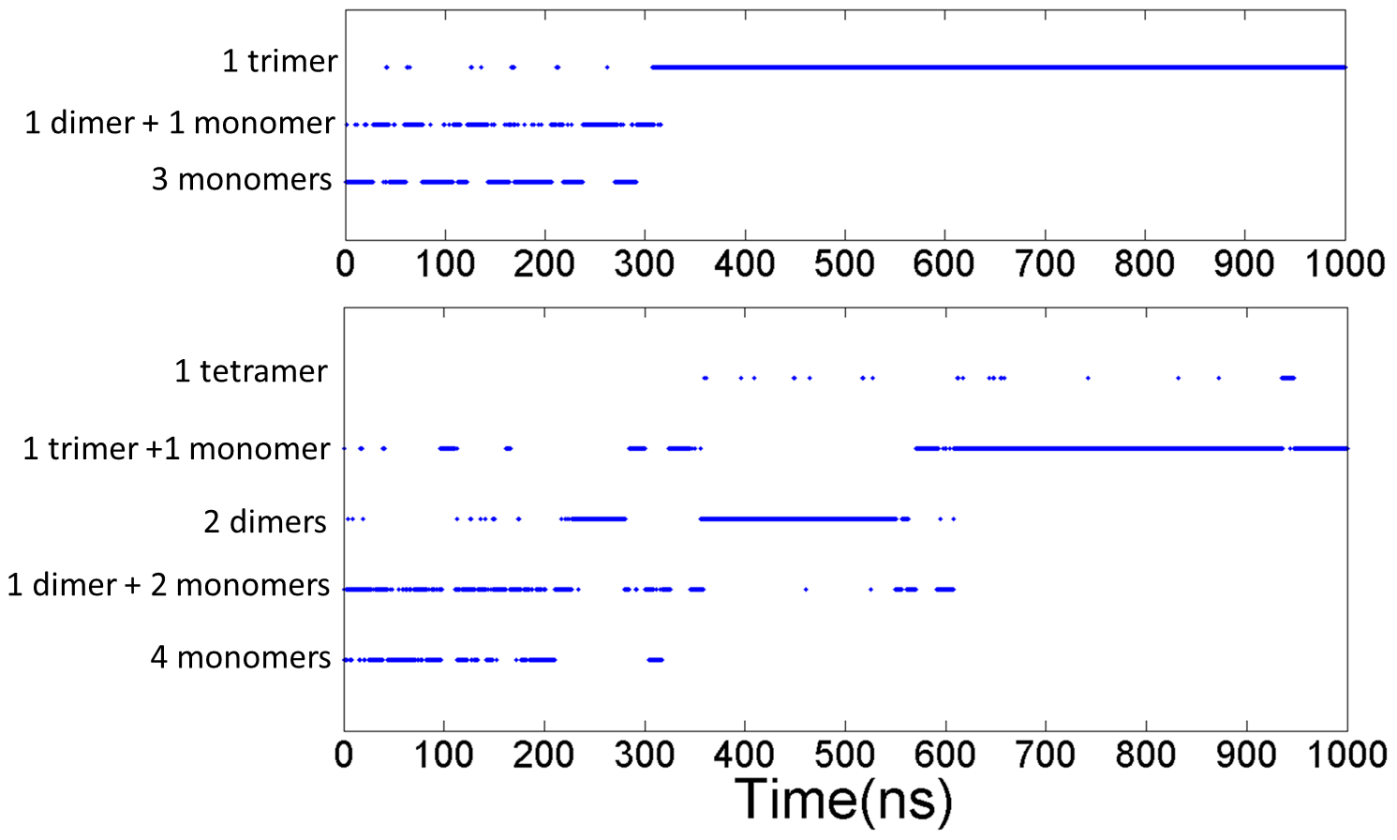
States of aggregation as a function of simulation time for the systems of (a) three peptides and (b) four peptides.

For the simulation of the three PrP106–126 peptides, from Fig. 11a, it can be seen that the three monomers were often separated with the state of 1 monomer and 1 dimer emerging frequently and the trimer distributed sparsely in the first 310 ns. After 310 ns, the three peptides reached a “stable state” and oligomerized into a trimer which existed until the ending of the simulation as the only state. In the simulations of the four monomers, the tetramer is hardly observed along the simulation time as illustrated in Fig. 11b. In first 360 ns, different states of the four monomers changed from one to another back and forth. Then, the state of 2 dimers was populated predominantly after 360 ns. The two dimers existed stably until 570 ns when one of the dimer dissociated. One of the isolated peptides quickly absorbed on the other dimer resulting in the 1 trimer and 1 monomer state. The trimer existed stably until 1000 ns. The two simulations both experienced persistent fluctuation before reaching “stable state”, which may indicated that association-dissociation of the peptides may be an obligatory step toward the formation of ordered oligomers.

**Figure 12:**
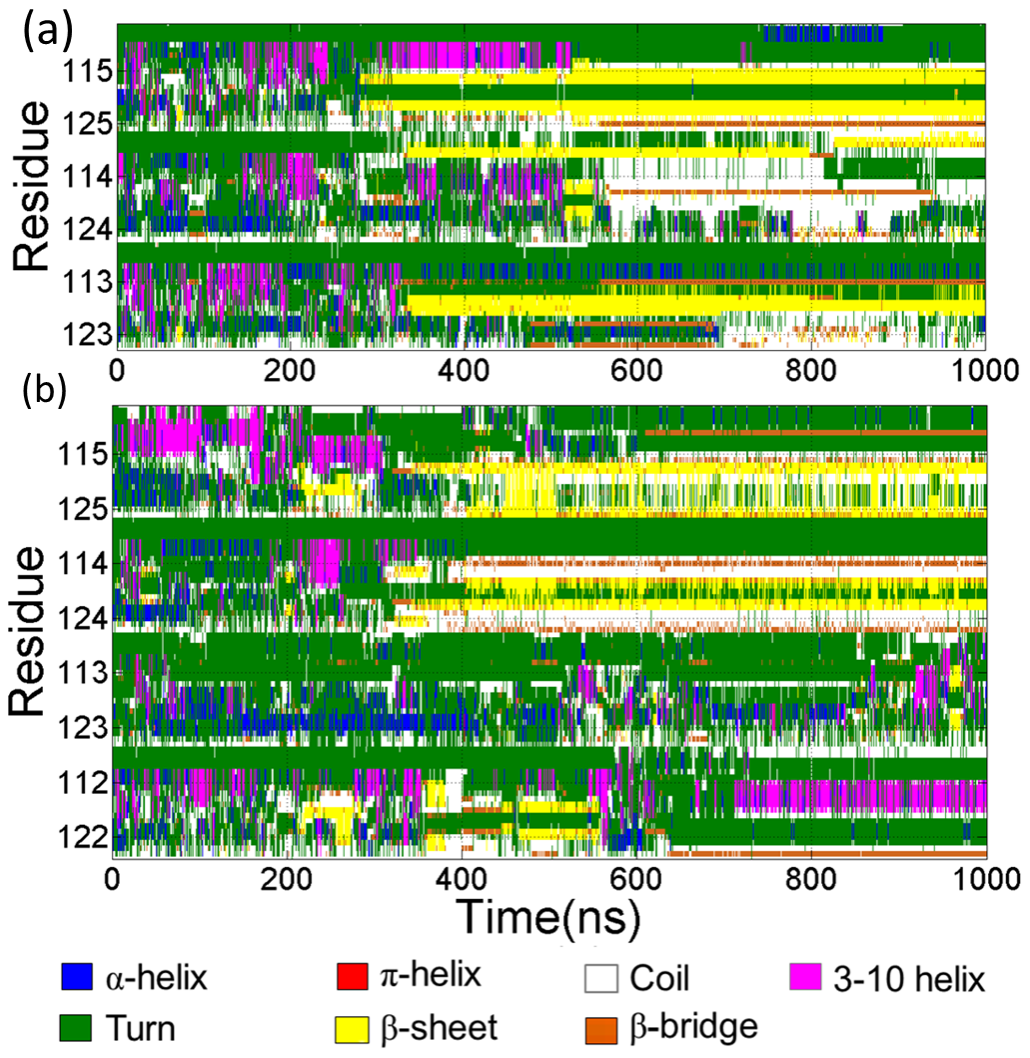
The evolution of secondary structure. (a) Three peptides in trimer forming simulation. From the top to bottom, Chain A, Chain B and Chain C were displayed in order. (b) four peptides in tetramer forming simulation. Chain A, Chain B, Chain C and Chain D are illustrated in order from top to bottom.

Fig. 12 showed the secondary structure evolutions during the simulations determined by STRIDE algorithm. In Fig. 12a, the secondary structures of the three peptides along the simulation time of the trimer formation were presented. In the first 270 ns, the three peptides display no well-defined secondary structures. The β-sheet ranging from residues 116 to residue 122 of Chain A appeared in about 270 ns then in about 330 ns the β-sheet structure occurred in the residues 109,110 of Chain B and shortly residues 115–119 of the third chain adopted β-sheet structure. Fig. 12b showed the time profile of secondary structures of the tetramer formation. Compared to the trimer formation, the secondary structure evolutions was more complex. In about 220 ns, β-sheet has begun to occur. The length of the β-sheet was not constant and changed in a certain range from residues 118 to residue 123 in Chain A and from residues 116 to 123 in Chain D respectively. Then the β-sheet disappeared in about 280 ns. In about 310 ns, the β-sheet formed again in the residue 115–123 of Chain B. Then Chain A and Chain D also adopted β-sheets subsequently. In about 550 ns, the β-sheet of Chain D was lost and the residues in C-terminus of Chain D participated in the formation of β-bridge after about 580 ns.

Based on the analysis of secondary structure evolutions and states of aggregation along simulation time, we found some interesting phenomena. For the simulations of the trimer and tetramer, there were predominantly populated β-hairpins in simulations, which corresponded to the stable status of aggregation. In contrast with the predominant coil structures of monomeric PrP106–126, the stable oligomers have a relatively higher β-sheet structure and lower helical structures.

Notably, the β-sheet structures are all located in the hydrophobic tails of PrP106–126 peptides. In Chain A of the three peptides in trimer formation simulations and Chain B of the four peptides in tetramer formation, the turns between the two short β-sheets formed in the identical region 118–120. Connecting the time that the hairpins formed in hydrophobic tails with residues 118–120 in their turns formed and the time when stable oligomers occurred, something interesting can be found. The stable trimer appeared (about 310 ns) after the formation of the hairpin in Chain A (about 270 ns)in the trimer formation simulations. The formation of trimer of the tetramer system was also after the formations of the hairpin in Chain B. it can be suggested that the behaviors of oligomerization of PrP106–126 and the formation of β-hairpins in the hydrophobic tail are closely related. The β-hairpins may play a significant role in the stabilization of the aggregates. Shea et al [25] also suggested the assembly of β-hairpin for oligomers of PrP106–126 using ion mobility spectrometry-mass spectrometry (IMS-MS) in conjunction with replica exchange molecular dynamics. However, Kuwata et al [24] have proposed a model for PrP106–126 fibrils with parallel beta-sheets and an uninterrupted stretch of 13 amino acids with beta-strand character. The discrepancy between the solid-state NMR of PrP106–126 fibrils and the simulations can be explained in following aspects. Firstly, the structural information obtained from solid-state NMR is ensemble averaged and reveals the property of bulk solution, which may contain multiple oligomeric and conformational states, while molecular dynamics simulations selectively examine monomeric and oligomeric states. Secondly, experimental conditions may cause the structural discrepancy. For example, Kuwata et al hydrated PrP106–126 in 100 mM sodium acetate, containing 150 mM NaCl (pH 5.5) and 50% acetonitrile, while molecular dynamics simulations were carried out in pure water. Reaction media may change the rate of aggregation and produce different aggregates. PrP fibrils may display polymorphsm due to kinetic or thermodynamics reasons.

Though the trimers sampled in the simulations of the trimer and tetramer formation were stable, we cannot rule out the possibility that the 1000 ns simulations are not fully converge because the formation of the amyloid fibril is a very slow process. However the “quasi-stable” states can provide important information about the process of oligomerization and the structural factors stabilizing the initial oligomers.

**Figure 13:**
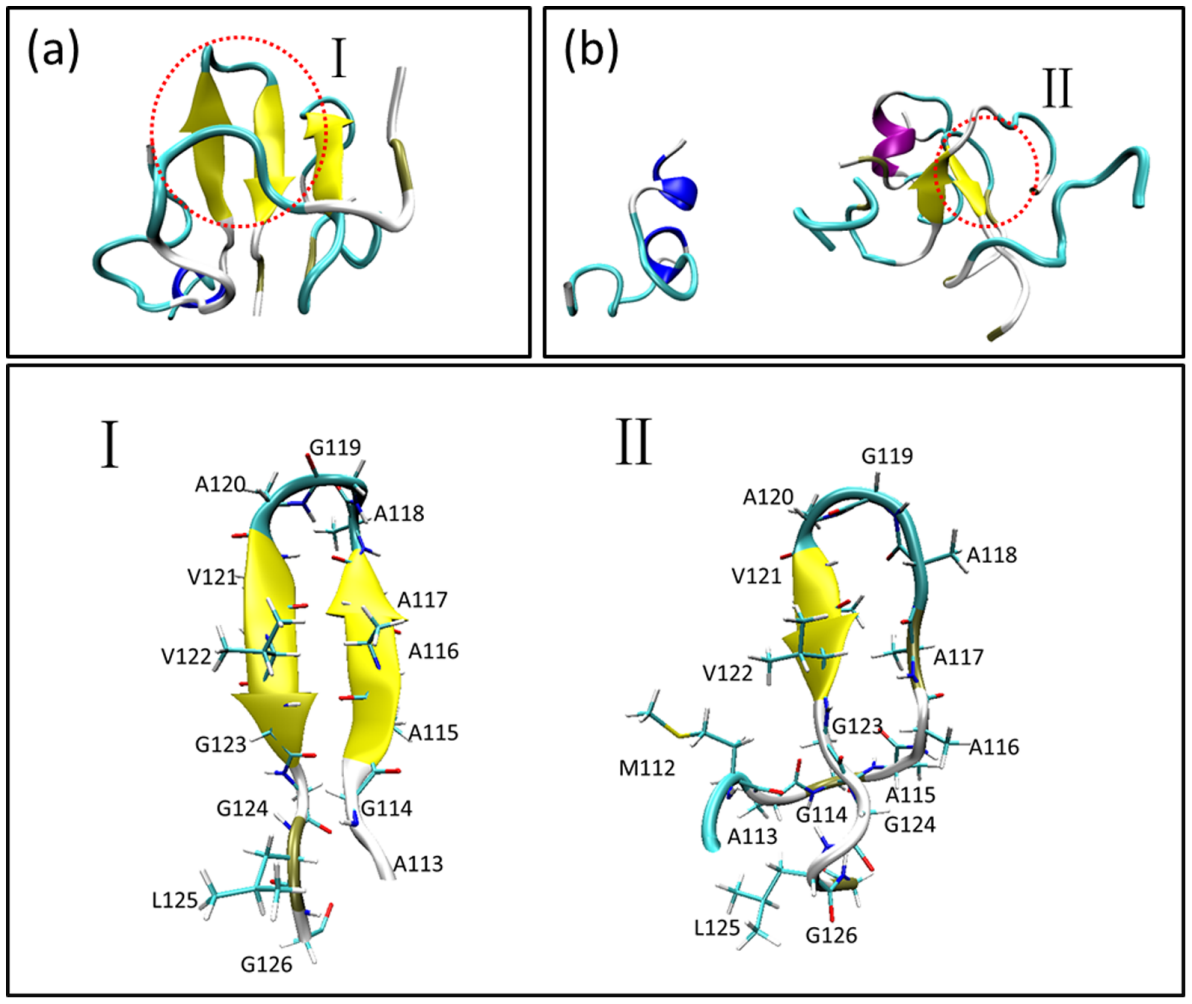
Last snapshots of the simulations and the detailed illustrations of β-hairpin acquired by the two stable trimers. The ending structures were colored based on secondary structure. The residues forming the β-hairpin were shown in Licorice and colored according to names of atoms.

**Figure 14:**
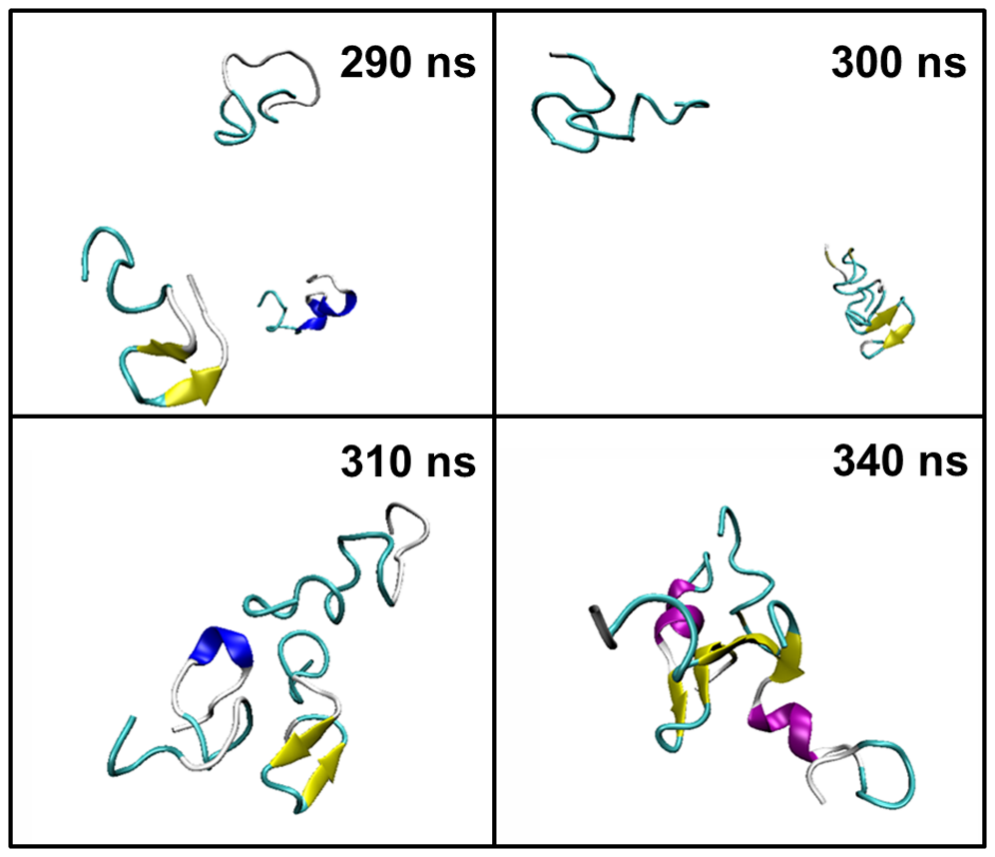
The forming process of the stable trimer formation.

**Figure 15.**
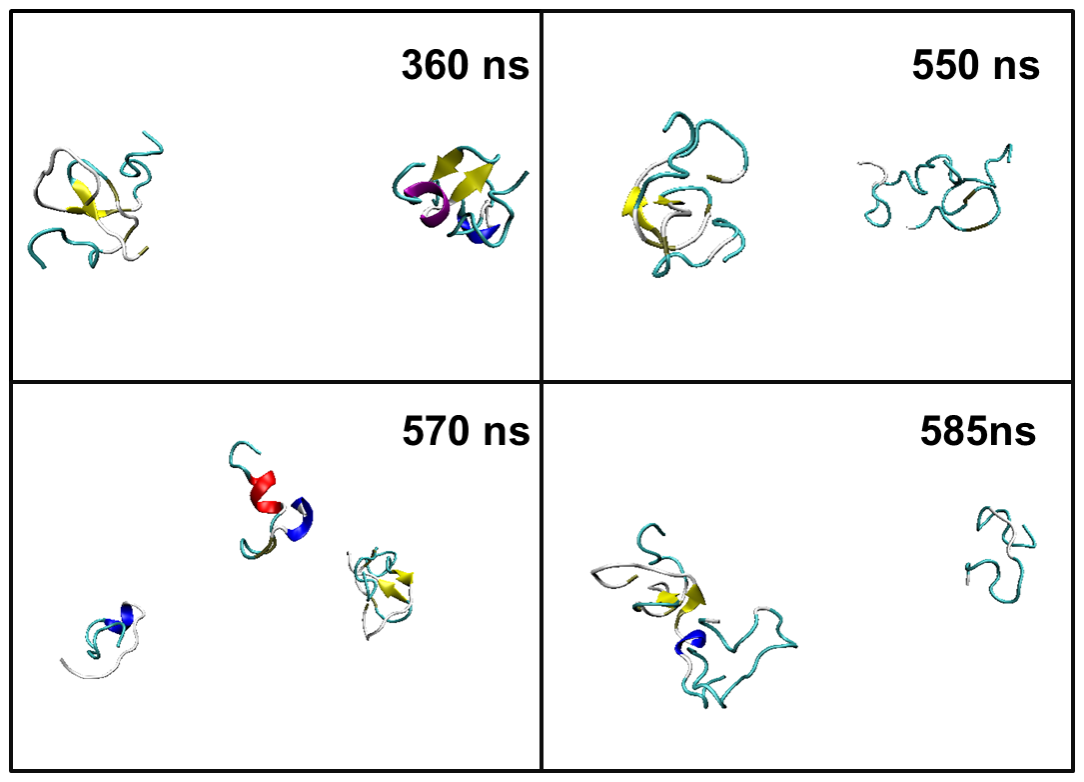
The process of oligomerization of the four PrP106–126 peptides.

Fig. 13 illustrated the last snapshots of the two systems. The stable trimers sampled from simulations contain similar C-terminal β-hairpin with residues 118–120 in turn. For the formation of the trimer as shown in Fig. 14, firstly the β-hairpin ranging from residues 115 to 123 in Chain A formed in about 270 ns, then, Chain B attached to this peptide with the β-hairpin structure forming stable dimer and Chain C gathered with the former dimer forming a trimer in about 310 ns. The orientations of the three peptides were adjusted and inter-chain β-sheet formed in about 360 ns. The trimer existed stably in the rest simulation time. The formation of trimer in the simulations of the four peptides was illustrated in Fig. 15. In about 360 ns, two dimers with β-haripin structure with residues 118–120 in turn formed and the structures were relatively stable. In about 550 ns, the β-hairpin included in one of the two dimers disappeared and then the dimer disassociated. One of the isolated peptides interacted with the existing dimer in about 580 ns resulting in a stable trimer.

This observation suggested that the hairpin structure in the hydrophobic region of PrP106–126 may play critical role in the initial oligomerization. The residues forming the hairpin structure include the most part of palindrome region residues 113–120. It has been reported that the palindrome region AGAAAAGA is necessary for both PrP^Sc^-like toxicity and fibril formation [81], [82]. Our study explained this phenomenon in atomic level.

## Conclusions

In this study, we carried out replica exchange molecular dynamics simulations and conventional molecular dynamics simulations in implicit solvent to study the structural features of PrP106–126 and the initial oligomerization of this peptide. The results of REMD reveal that monomeric PrP206–126 is not well-structured, with a high tendency to form turn and coil structure. Dimeric PrP106–126 also displayed structural diversity and hydrophobic interaction drove the dimerization. The simulations of the trimer formation and tetramer formation “converged” to relative stable oligomers. These oligomers shared the similar structure element β-hairpin in the hydrophobic tail of PrP106–126 which may promote oligomerization and stabilize oligomers. The study may help in developing new approaches toward rational drug design to block amyloid formation.
